# Supplementary material for: Resolving the full spectrum of human genome variation using Linked-Reads
Source: Genome Res. 2019 Apr;29(4):635–45. doi: 10.1101/gr.234443.118 (PMC6442396; doi:10.1101/gr.234443.118)
Supplement: Supplemental Material [file supp_gr.234443.118_Supplemental_longranger-2.2.zip › longranger-2.2.tar.bz2!/lr2.2-public/lib/stan/cmdstan/stan/doc/api/html/classstan_1_1mcmc_1_1base__hmc.html]

Stan: stan::mcmc::base\_hmc< M, P, H, I, BaseRNG > Class Template Reference


|  |  |
| --- | --- |
|  | Stan  2.5.0  probability, sampling & optimization |


- Main Page
- Related Pages
- Namespaces
- Classes
- Files

- Class List
- Class Index
- Class Hierarchy
- Class Members

All Classes Namespaces Files Functions Variables Typedefs Enumerations Enumerator Friends Macros Pages

- stan
- mcmc
- base\_hmc

Public Member Functions |
Protected Attributes |
List of all members

stan::mcmc::base\_hmc< M, P, H, I, BaseRNG > Class Template Reference

`#include <base_hmc.hpp>`

Inheritance diagram for stan::mcmc::base\_hmc< M, P, H, I, BaseRNG >:

|  |  |
| --- | --- |
| Public Member Functions | |
|  | base\_hmc (M &m, BaseRNG &rng, std::ostream \*o, std::ostream \*e) |
|  | |
| void | write\_sampler\_state (std::ostream \*o) |
|  | |
| void | get\_sampler\_diagnostic\_names (std::vector< std::string > &model\_names, std::vector< std::string > &names) |
|  | |
| void | get\_sampler\_diagnostics (std::vector< double > &values) |
|  | |
| void | seed (const Eigen::VectorXd &q) |
|  | |
| void | init\_stepsize () |
|  | |
| P & | z () |
|  | |
| virtual void | set\_nominal\_stepsize (const double e) |
|  | |
| double | get\_nominal\_stepsize () |
|  | |
| double | get\_current\_stepsize () |
|  | |
| virtual void | set\_stepsize\_jitter (const double j) |
|  | |
| double | get\_stepsize\_jitter () |
|  | |
| void | sample\_stepsize () |
|  | |
| Public Member Functions inherited from stan::mcmc::base\_mcmc | |
|  | base\_mcmc (std::ostream \*o, std::ostream \*e) |
|  | |
| virtual | ~base\_mcmc () |
|  | |
| virtual sample | transition (sample &init\_sample)=0 |
|  | |
| std::string | name () |
|  | |
| virtual void | write\_sampler\_param\_names (std::ostream &o) |
|  | |
| virtual void | write\_sampler\_params (std::ostream &o) |
|  | |
| virtual void | get\_sampler\_param\_names (std::vector< std::string > &names) |
|  | |
| virtual void | get\_sampler\_params (std::vector< double > &values) |
|  | |

|  |  |
| --- | --- |
| Protected Attributes | |
| P | z\_ |
|  | |
| I< H< M, BaseRNG >, P > | integrator\_ |
|  | |
| H< M, BaseRNG > | hamiltonian\_ |
|  | |
| BaseRNG & | rand\_int\_ |
|  | |
| boost::uniform\_01< BaseRNG & > | rand\_uniform\_ |
|  | |
| double | nom\_epsilon\_ |
|  | |
| double | epsilon\_ |
|  | |
| double | epsilon\_jitter\_ |
|  | |
| Protected Attributes inherited from stan::mcmc::base\_mcmc | |
| std::string | name\_ |
|  | |
| std::ostream \* | out\_stream\_ |
|  | |
| std::ostream \* | err\_stream\_ |
|  | |

## Detailed Description

### template<class M, class P, template< class, class > class H, template< class, class > class I, class BaseRNG> class stan::mcmc::base\_hmc< M, P, H, I, BaseRNG >

Definition at line 20 of file base\_hmc.hpp.

## Constructor & Destructor Documentation

template<class M, class P, template< class, class > class H, template< class, class > class I, class BaseRNG>

|  |  |  |  |
| --- | --- | --- | --- |
| stan::mcmc::base\_hmc< M, P, H, I, BaseRNG >::base\_hmc | ( | M & | *m*, |
|  |  | BaseRNG & | *rng*, |
|  |  | std::ostream \* | *o*, |
|  |  | std::ostream \* | *e* |
|  | ) |  |  |

inline

Definition at line 24 of file base\_hmc.hpp.

## Member Function Documentation

template<class M, class P, template< class, class > class H, template< class, class > class I, class BaseRNG>

|  |  |  |  |  |
| --- | --- | --- | --- | --- |
| double stan::mcmc::base\_hmc< M, P, H, I, BaseRNG >::get\_current\_stepsize | ( |  | ) |  |

inline

Definition at line 117 of file base\_hmc.hpp.

template<class M, class P, template< class, class > class H, template< class, class > class I, class BaseRNG>

|  |  |  |  |  |
| --- | --- | --- | --- | --- |
| double stan::mcmc::base\_hmc< M, P, H, I, BaseRNG >::get\_nominal\_stepsize | ( |  | ) |  |

inline

Definition at line 115 of file base\_hmc.hpp.

template<class M, class P, template< class, class > class H, template< class, class > class I, class BaseRNG>

|  |  |  |  |
| --- | --- | --- | --- |
| void stan::mcmc::base\_hmc< M, P, H, I, BaseRNG >::get\_sampler\_diagnostic\_names | ( | std::vector< std::string > & | *model\_names*, |
|  |  | std::vector< std::string > & | *names* |
|  | ) |  |  |

inlinevirtual

Reimplemented from stan::mcmc::base\_mcmc.

Definition at line 42 of file base\_hmc.hpp.

template<class M, class P, template< class, class > class H, template< class, class > class I, class BaseRNG>

|  |  |  |  |  |  |
| --- | --- | --- | --- | --- | --- |
| void stan::mcmc::base\_hmc< M, P, H, I, BaseRNG >::get\_sampler\_diagnostics | ( | std::vector< double > & | *values* | ) |  |

inlinevirtual

Reimplemented from stan::mcmc::base\_mcmc.

Definition at line 47 of file base\_hmc.hpp.

template<class M, class P, template< class, class > class H, template< class, class > class I, class BaseRNG>

|  |  |  |  |  |
| --- | --- | --- | --- | --- |
| double stan::mcmc::base\_hmc< M, P, H, I, BaseRNG >::get\_stepsize\_jitter | ( |  | ) |  |

inline

Definition at line 123 of file base\_hmc.hpp.

template<class M, class P, template< class, class > class H, template< class, class > class I, class BaseRNG>

|  |  |  |  |  |
| --- | --- | --- | --- | --- |
| void stan::mcmc::base\_hmc< M, P, H, I, BaseRNG >::init\_stepsize | ( |  | ) |  |

inline

Definition at line 55 of file base\_hmc.hpp.

template<class M, class P, template< class, class > class H, template< class, class > class I, class BaseRNG>

|  |  |  |  |  |
| --- | --- | --- | --- | --- |
| void stan::mcmc::base\_hmc< M, P, H, I, BaseRNG >::sample\_stepsize | ( |  | ) |  |

inline

Definition at line 125 of file base\_hmc.hpp.

template<class M, class P, template< class, class > class H, template< class, class > class I, class BaseRNG>

|  |  |  |  |  |  |
| --- | --- | --- | --- | --- | --- |
| void stan::mcmc::base\_hmc< M, P, H, I, BaseRNG >::seed | ( | const Eigen::VectorXd & | *q* | ) |  |

inline

Definition at line 51 of file base\_hmc.hpp.

template<class M, class P, template< class, class > class H, template< class, class > class I, class BaseRNG>

|  |  |  |  |  |  |
| --- | --- | --- | --- | --- | --- |
| virtual void stan::mcmc::base\_hmc< M, P, H, I, BaseRNG >::set\_nominal\_stepsize | ( | const double | *e* | ) |  |

inlinevirtual

Reimplemented in stan::mcmc::base\_static\_hmc< M, P, H, I, BaseRNG >, stan::mcmc::base\_static\_hmc< M, diag\_e\_point, diag\_e\_metric, expl\_leapfrog, BaseRNG >, stan::mcmc::base\_static\_hmc< M, dense\_e\_point, dense\_e\_metric, expl\_leapfrog, BaseRNG >, and stan::mcmc::base\_static\_hmc< M, unit\_e\_point, unit\_e\_metric, expl\_leapfrog, BaseRNG >.

Definition at line 111 of file base\_hmc.hpp.

template<class M, class P, template< class, class > class H, template< class, class > class I, class BaseRNG>

|  |  |  |  |  |  |
| --- | --- | --- | --- | --- | --- |
| virtual void stan::mcmc::base\_hmc< M, P, H, I, BaseRNG >::set\_stepsize\_jitter | ( | const double | *j* | ) |  |

inlinevirtual

Definition at line 119 of file base\_hmc.hpp.

template<class M, class P, template< class, class > class H, template< class, class > class I, class BaseRNG>

|  |  |  |  |  |  |
| --- | --- | --- | --- | --- | --- |
| void stan::mcmc::base\_hmc< M, P, H, I, BaseRNG >::write\_sampler\_state | ( | std::ostream \* | *o* | ) |  |

inlinevirtual

Reimplemented from stan::mcmc::base\_mcmc.

Definition at line 36 of file base\_hmc.hpp.

template<class M, class P, template< class, class > class H, template< class, class > class I, class BaseRNG>

|  |  |  |  |  |
| --- | --- | --- | --- | --- |
| P& stan::mcmc::base\_hmc< M, P, H, I, BaseRNG >::z | ( |  | ) |  |

inline

Definition at line 109 of file base\_hmc.hpp.

## Member Data Documentation

template<class M, class P, template< class, class > class H, template< class, class > class I, class BaseRNG>

double stan::mcmc::base\_hmc< M, P, H, I, BaseRNG >::epsilon\_

protected

Definition at line 143 of file base\_hmc.hpp.

template<class M, class P, template< class, class > class H, template< class, class > class I, class BaseRNG>

double stan::mcmc::base\_hmc< M, P, H, I, BaseRNG >::epsilon\_jitter\_

protected

Definition at line 144 of file base\_hmc.hpp.

template<class M, class P, template< class, class > class H, template< class, class > class I, class BaseRNG>

H<M, BaseRNG> stan::mcmc::base\_hmc< M, P, H, I, BaseRNG >::hamiltonian\_

protected

Definition at line 135 of file base\_hmc.hpp.

template<class M, class P, template< class, class > class H, template< class, class > class I, class BaseRNG>

I<H<M, BaseRNG>, P> stan::mcmc::base\_hmc< M, P, H, I, BaseRNG >::integrator\_

protected

Definition at line 134 of file base\_hmc.hpp.

template<class M, class P, template< class, class > class H, template< class, class > class I, class BaseRNG>

double stan::mcmc::base\_hmc< M, P, H, I, BaseRNG >::nom\_epsilon\_

protected

Definition at line 142 of file base\_hmc.hpp.

template<class M, class P, template< class, class > class H, template< class, class > class I, class BaseRNG>

BaseRNG& stan::mcmc::base\_hmc< M, P, H, I, BaseRNG >::rand\_int\_

protected

Definition at line 137 of file base\_hmc.hpp.

template<class M, class P, template< class, class > class H, template< class, class > class I, class BaseRNG>

boost::uniform\_01<BaseRNG&> stan::mcmc::base\_hmc< M, P, H, I, BaseRNG >::rand\_uniform\_

protected

Definition at line 140 of file base\_hmc.hpp.

template<class M, class P, template< class, class > class H, template< class, class > class I, class BaseRNG>

P stan::mcmc::base\_hmc< M, P, H, I, BaseRNG >::z\_

protected

Definition at line 133 of file base\_hmc.hpp.

---

The documentation for this class was generated from the following file:

- src/stan/mcmc/hmc/base\_hmc.hpp


---

[ Stan Home Page ]

*© 2011–2014,
Stan Development Team.*
